# Psychosocial impacts of Baby Friendly Spaces for Rohingya refugee mothers in Bangladesh: A pragmatic cluster-randomized controlled trial

**DOI:** 10.1017/gmh.2024.58

**Published:** 2024-05-07

**Authors:** Amanda J. Nguyen, Sarah M. Murray, Kh Shafiur Rahaman, Molly E. Lasater, Suzit Barua, Catherine Lee, Matthew Schojan, Brigitte Tonon, Laetitia Clouin, Karine Le Roch

**Affiliations:** 1 Department of Human Services, School of Education and Human Development, University of Virginia, Charlottesville, VA, USA; 2 Department of Mental Health, Johns Hopkins Bloomberg School of Public Health, Baltimore, MD, USA; 3 Action Against Hunger, Cox’s Bazar, Bangladesh; 4 Action contre La Faim, Paris, France

**Keywords:** psychosocial, mental health, maternal, infants, refugee, nutrition, Rohingya, Bangladesh

## Abstract

**Background:**

This study evaluated the effectiveness of Baby Friendly Spaces (BFS), a psychosocial support program for Rohingya refugee mothers of malnourished young children in Bangladesh. Because BFS was already being implemented, we examined the benefit of enhancing implementation supports.

**Methods:**

In matched pairs, 10 sites were randomized to provide BFS treatment as usual (BFS-TAU) or to receive enhanced implementation support (BFS-IE). 600 mothers were enrolled and reported on maternal distress, functional impairment, subjective well-being and coping at baseline and 8-week follow-up. Data were analyzed using multilevel linear regression models to account for clustering; sensitivity analyses adjusted for the small number of clusters.

**Results:**

Significant within-group improvements in BFSIE were observed for distres (−.48, p = .014), functional impairment (−.30, p = .002) and subjective well-being (.92, p = .011); improvements in BFS-TAU were smaller and not statistically significant. Between-group comparisons favored BFS-IE for distress (β = −.30, p = .058) and well-being (β = .58, p = .038). Sensitivity adjustments produced p-values above .05 for all between-group comparisons.

**Discussion:**

Feasible adjustments to implementation can improve program delivery to increase impact on maternal distress and well-being. Although results should be interpreted with caution, study design limitations are common in pragmatic, field-based research.

## Impact statement

As maternal mental health and child health are clearly linked, supporting maternal mental health and well-being is critical to the promotion of child development and care practices that can ultimately promote positive child health outcomes. Addressing maternal mental health is particularly critical in settings of adversity where exposure to traumatic events, deprivation and ongoing stressors threaten the well-being of both caregivers and child. However, scant research addresses how to support the integration of evidence-based mental health and psychosocial support (MHPSS) services into other care settings, such as child nutrition. Conducting rigorous research to grow this evidence is complicated in real-world humanitarian settings where logistical challenges and ethical constraints render some research designs infeasible. This paper presents a study designed to evaluate the impact of Baby Friendly Spaces (BFS), a psychosocial support program for mothers of malnourished young children, delivered in Rohingya refugee camps in Cox’s Bazar, Bangladesh. In this study, mothers and babies participated in BFS as part of a broader package of child nutrition care. Because all operating Integrated Nutrition Centers offered BFS, it was not possible to randomize participants or centers to treatment and control conditions. Instead, we provided additional implementation support in half of the centers to determine the added value of improving existing programming and found that doing so improved the mental health impacts for mothers in the program. This study adds to a growing body of research in humanitarian settings showcasing not only the value of integrating psychosocial programming into basic health services, but also the necessity of providing ongoing supervision and support to ensure that such programming has optimal impact among participants.

## Introduction

Globally, the connections between maternal well-being and child morbidity and mortality are well established. Maternal depression has been associated with poor nutrition outcomes, including being underweight or stunted (Surkan et al., 2011), and it has been identified as a key factor to target in efforts to ensure that children under 5 years of age reach their full developmental potential in low-income settings (Ruel and Alderman, 2013; Surkan et al., 2016). Research has increasingly pointed to a critical interrelationship between maternal depression, mother–child bonding and child nutrition and morbidity, specifically within conflict-affected settings (Maternal and Child Nutrition Study Group, 2013; Tol et al., 2013), where exposure to potentially traumatic events substantially increases the risk of psychosocial distress and associated functional impairment among adults (Steel et al., 2009). Young children are a particularly critical population to protect in conflict-related displacement because of their vulnerability to infectious diseases, and the potential long-term effects of illnesses in this period on nutrition and developmental trajectories (Toole and Waldman, 1997; Bendavid et al., 2021; Clark, 2021).

Accordingly, in a recent consensus-based research agenda-building exercise for psychosocial support (PSS) programming, stakeholders pointed to a particular need for evaluations of community-based programs that integrate delivery of MHPSS into other routine health services (*e.g.*, nutritional support) and that target caregivers to improve child well-being (Lee et al., 2019). In general, there is a relative lack of evidence for more prevention-oriented or low-intensity psychosocial programs aimed at improving or promoting mental health in humanitarian contexts in low- and middle-income countries, as demonstrated by an extensive systematic literature review (Haroz et al., 2020). Yet, few parent–child integrated PSS programs exist that aim to prevent or respond to these issues (Ruel and Alderman, 2013; Surkan et al., 2016). Given their promise as a sustainable approach to improving the psychosocial well-being of both target groups (Daelmans et al., 2021), there is a clear need to improve evidence-based programming and practical, operational guidance for implementation in humanitarian settings (Bhutta et al., 2021).

Critically, there is strong empirical support for the link between the quality of implementation of programs and health outcomes in prevention and promotion research (Durlak and DuPre, 2008). For instance, prior effectiveness studies of the child friendly spaces program, which is commonly implemented in humanitarian contexts, have implicated service quality as an important driver of heterogeneity in impact (Hermosilla et al., 2019). Similar mixed findings on the impacts of psychological interventions on maternal mental health have elucidated challenges with low fidelity in intervention delivery and highlighted the need for more careful attention to how providers are trained and how nonspecific therapeutic competencies are taught and supported (Gorman et al., 2021). Provider competency is key to program fidelity and service quality and is insufficiently supported by training in the absence of ongoing supervision (Beidas, Bond). Yet contextual and organizational challenges in humanitarian settings often result in the neglect of ongoing supervision for community-based MHPSS (Perera et al., 2021). This gap illustrates why MHPSS workforce strengthening, service integration and supervision have been identified as top MHPSS research priorities (Tol et al., 2023).

One program that does exist for integration of maternal mental health and child nutrition in humanitarian settings is Action Against Hunger’s (Action contre la Faim, ACF) Baby Friendly Spaces (BFS): a holistic program designed to enhance mothers’ well-being to buffer against the deleterious health and developmental impacts of conflict and disaster on children (ACF, 2014). ACF began implementing BFS for pregnant and lactating mothers of children under the age of 2 years in humanitarian emergencies in 2006 to prevent child undernutrition and reduce child morbidity and mortality. BFS is designed to be flexible to meet the unique needs of different conflict-affected populations in a community-based approach, but always focuses on two domains: PSS to improve maternal well-being and childcare practices that target caregiver functioning to also address child well-being and development. In so doing, BFS strengthens mothers’ internal resources and skills in caring for their children to positively impact their nutritional status and well-being of their children during humanitarian emergencies.

A prior process evaluation of the BFS program in Ethiopia found some positive changes among mothers enrolled in the program (Lasater et al., 2020), and a single-group evaluation of the effect of a 3-month maternal PSS group implemented by ACF among pregnant Rohingya women in Cox’s Bazar found changes in maternal well-being and childcare knowledge (Corna et al., 2019). However, no controlled study of the BFS program has been done to date. Therefore, to assess the effectiveness of this program and address the broader gap in knowledge on how to address maternal mental health and child health in an integrated way in humanitarian settings, we aimed to conduct a cluster-randomized controlled trial of the BFS program as implemented in Cox’s Bazar, Bangladesh to Rohingya refugee mothers. As the program was already being offered at all ACF sites, randomization to a nonintervention control was not feasible. Instead, consistent with the literature above linking implementation quality to program outcomes and the reality that the BFS program was being implemented with relatively little structure, we hypothesized that additional training, supervision and support to standardize and improve quality of delivery would improve outcomes for mothers relative to delivery of the BFS program “as usual.”

## Methods

### Study setting and design

This study was conducted in ACF-operated Integrated Nutrition Centers (INCs) in Rohingya refugee camps in Cox’s Bazar, Bangladesh in 2021–2022. The Rohingya people have experienced decades of discrimination and persecution in Eastern Myanmar, with periodic spikes in violence over decades leading to recurrent displacement of Rohingyas into Bangladesh. In 2017, nearly 700,000 Rohingya fled into Cox’s Bazar in response to an escalation of violence (UNICEF, 2019a). Refugees in Cox’s Bazar remain highly dependent on aid due to restrictions on employment in the camps, and are living in overcrowded, difficult conditions with serious impacts on health and well-being (UNICEF, 2019b). A previous needs assessment found that approximately one-third of all adults screened in ACF services reported extreme levels of stress; half of adults who underwent a full psychosocial evaluation reported suicidal ideation (ACF, 2016, 2017); and in just the first half of 2019, nearly 9,000 children were treated for severe acute malnutrition by ACF (UNICEF, 2019a).

Aided by a presence in the region dating back to 2006, at the time of this study, ACF operated 14 INCs across the Ukhiya and Teknaf areas, each with a BFS program that completed on average between 50 and 100 intakes per month of mother–child dyads in which the child was experiencing malnourishment and receiving nutritional treatment referred from the outpatient therapeutic feeding program. The two areas are generally comparable with the exception of length of time refugees had been living in Bangladesh, and we prioritized INCs with catchment areas that predominantly hosted Rohingya refugees from the 2017 influx. Therefore, out of the 14 existing ACF INCs, 10 were included in the study as three were located in areas that were primarily home to refugees who arrived before the mass displacement of Rohingya in 2017 and might differ substantially from more recent arrivals; the remaining site was unmatched and so excluded for balance.

We sought to compare maternal psychosocial outcomes between the two conditions using a pragmatic cluster-randomized trial design. In the comparison condition (*i.e.*, BFS Treatment as Usual (BFS-TAU); K = 5), INCs continued to deliver BFS activities as they were currently being offered. In the active study condition (*i.e.*, BFS Implementation-Enhanced (BFS-IE)), the BFS program was standardized, and BFS staff were retrained and continued to receive additional supervision and implementation supports throughout the study period. In both conditions, maternal-child dyads were recruited at BFS intake and completed a baseline assessment, and then participated in BFS according to usual programming before completing a follow-up assessment 8–10 weeks later. The study was registered on ClinicalTrials.gov (identifier: NCT05281575).

### Randomization

INCs were randomized on a 1:1 allocation ratio within pairs matched by location (Ukhiya or Teknaf), camp number and psychologist. Specifically, whereas six of the sites each had their own center-based psychologists, the other four sites shared two psychologists (each supporting two sites). In those cases, because the psychologist worked closely with the BFS program, INCs with a shared psychologist were randomized together. This resulted in four matched pairs for randomization (Table 1). We then generated a random number sequence, allocating the INC with the lower of the two numbers in each pair to the intervention group. Using this approach, AJN generated five randomization sequence options, one of which was then randomly selected from a hat by KLR. This approach allowed members of the team without personal connection to any of the sites to generate the allocation sequence while remaining blinded.

**Table 1.** INC matched pairs for randomization

INC	Location	Camp	BFS start date	No. psychologist	Matched pair
1E–01	Ukhiya	1E	2021	1^a^	1
1E–02	Ukhiya	1E	2020		
3–1	Ukhiya	3	2018	1^a^	1
3.2	Ukhiya	3	2021		
1W–1	Ukhiya	1W	2018	1	2
1W–2	Ukhiya	1W	2018	1	2
2W	Ukhiya	2W	2018	1	3
C21	Ukhiya	21	2018	1	3
C24	Teknaf	24	2018	1	4
C26	Teknaf	26	2018	1	4

a Sites were randomized as a pair due to their sharing a single psychologist.

### Participants

At the time of their intake into the BFS program, 600 Rohingya mother–child dyads were recruited across the 10 participating INCs, aiming for n = 300 in each study condition. Study eligibility criteria included being an adult (age 18 or older) Rohingya mother of a child under 2 years of age identified as experiencing moderate or severe acute malnutrition without complication by ACF and enrolling for the first time in BFS services at a participating INC. If a mother had more than one qualifying child, the youngest eligible child was identified as the index child for the study due to a BFS focus on breastfeeding supports. The designation of a single index child for the study did not, however, preclude the mother from participating in programmatic activities for all children. Study exclusion criteria included mothers who were planning to leave the area within the 2-month intervention and follow-up period, mothers with cognitive impairment or psychosis that would preclude participation in program activities, as well as mothers of children with severe developmental disabilities or severe malnutrition with complications. Additionally, anyone receiving referral care outside ACF for more severe mental health or protection needs (in accordance with standard ACF protocols) were excluded. Women who were deemed ineligible for the study were still able to enroll in the program and receive all available services that they would be regularly eligible for.

### Interventions

In support of maternal psychosocial well-being and childcare practices, the following activities are typically delivered in BFS, either individually or in groups, by trained psychosocial workers: breastfeeding counseling; parenting skills; mother–child bonding activities that provide physical and neurocognitive stimulation; hygiene promotion and maternal PSS (*i.e.*, psychoeducation, stress management). To be enrolled, mothers receive an initial assessment with a trained psychosocial worker accompanied by a Rohingya volunteer using standardized instruments to identify specific psychosocial and care practice needs. Based on this initial assessment, the psychosocial worker refers women to scheduled BFS activities and/or arranges home visits as needed for monitoring and follow-up. The BFS also serves as a point of referral to other services (*e.g.,* higher-level mental health care, gender-based violence services, *etc.*). The program is designed so that women can drop in and attend BFS activities as they desire, but they are encouraged to attend at least weekly alongside nutrition appointments for the malnourished child (which typically take place weekly, but were reduced to monthly or twice monthly during the COVID-19 pandemic).

While the use of a non-BFS control condition would provide stronger evidence for the full impact of BFS, this was neither ethical nor feasible as all INCs were already offering the program. However, due to COVID-19-related staffing and training interruptions as well as a lack of intervention standardization, at the time of this study, BFS activities in Cox’s Bazar had experienced a natural drift in fidelity and were operating as largely unstructured, nonspecific recreational spaces where children were able to play either alongside mothers and BFS staff or while mothers participated in other program activities. In addition, whereas providers received general organizational oversight, they were not regularly participating in clinical supervision or given additional intervention materials to ensure BFS intervention fidelity or to promote core competencies in program delivery. With the exception of training updates related to the use of a new intake assessment, this state of implementation constituted the active comparison condition, that is, BFS-TAU. We anticipated that the program offered in this format would still be generally supportive but not optimized to impact priority outcomes.

Given its potential lessons for practice, a detailed description of our approach to developing the implementation-enhanced BFS condition (*i.e.*, BFS-IE) is separately available (Le Roch et al., n.d.). A summary of the two study conditions is also provided in Table 2. Briefly, in this condition, an MHPSS expert consultant worked with ACF to balance the need for program flexibility while creating an updated curriculum of standardized activities that prioritized five group activities: psychostimulation integrated in nutrition, in free baby play, through breastfeeding practices, in baby massage and hygiene and family support. Each of these activities followed a standard structure and was accompanied by brief guidance sheets. This program standardization facilitated targeted training and purpose-driven, programmatically aligned supervision to improve intervention quality (Kendall and Frank, 2018). Psychosocial workers and psychologists in the BFS-IE condition were retrained using these newly collaboratively developed materials over six half-day training sessions and two follow-up refresher trainings, all with a focus on building core therapeutic and self-care skills. The curricula were culturally and contextually adapted throughout the training. Staff in the enhanced arm then received biweekly group supervision over the course of the study, provided by the same expert consultant. The supervision structure also followed a standard format that mirrored the format of BFS facilitation and focused on BFS activities, discussion and self-care. Consistent with the literature reviewed above, our *implementation* theory of change was that by providing updated training and ongoing clinical supervision, provider competency and intervention fidelity would be improved, resulting in more psychosocial benefit to clients (McBride and Travers, 2023).

**Table 2.** Description of study conditions

Implementation support	BFS-TAU	BFS-IE
Intervention activities	Program audit determined the program was operating largely as provision of unstructured, nonspecific supports around play with children	Developed five priority activities, with standard structure and accompanying guidance sheet and fidelity checklist, contextualized for Cox’s Bazar to promote psychostimulation in: nutrition, free baby play, breastfeeding practices, baby massage and hygiene and family support
Training for psychosocial workers and psychologists	No retraining; original training conducted at least 3 years prior and not well documented, though was based on ACF’s standard BFS manual which was not contextualized for delivery in Cox’s Bazar	18 h of retraining using a manualized training curriculum focused on developing and strengthening staff capacities to deliver the newly structured priority activities Two follow–up sessions focused on the use of learnings in practice, changes observed and troubleshooting challenges faced
Supervision of psychosocial workers’ day–to–day activities	Provided by on–site psychologists but with no documentation and unclear roles; seemingly focused on general management and periodic crisis consultation	Case management meetings held bi–weekly by on–site psychologist
Clinical supervision	No supplemental supervision activities beyond what was described above	Conducted remotely by MHPSS consultant twice per month, following a standard format which mirrored the format of BFS facilitation and focused on BFS program activities, fidelity and self–care

### Study procedures

Women were screened for study eligibility by BFS providers during their program intake. Eligible participants were informed of the study and those who indicated an interest in study participation were referred to a data collector team consisting of a trained, Rohingya-speaking Bengali data collector accompanied by a Rohingya volunteer. Because of geographic constraints, each of the 10 data collection teams had a primary center assignment, although they periodically rotated to provide coverage at other centers when needed. All consent and data collection conversations were conducted in the Rohingya language, with Rohingya community volunteers assisting the Chittagonian Bengali speaking staff.

Data collectors obtained oral informed consent and administered the assessment interview, typically during the same intake appointment. Baseline data collection lasted between 1 and 2 h and was completed prior to the participant engaging in any BFS program activities. Data were collected *via* handheld tablet using KoBo Collect/Toolbox (KoBo Inc., 2019). As consent was obtained at the same time as the interview, consent was also documented in Kobo Collect/Toolbox. Participants were then free to attend BFS activities as usual. All sites recorded detailed information (as part of regular program monitoring) regarding attendance and activities delivered. Two trained observers rotated through INCs to record intervention fidelity according to a study-developed checklist (see Le Roch et al., n.d. for more details). Follow-up assessments were completed 8 weeks after baseline using the same assessment and approach, with interviews conducted either at the INCs or at the participants’ homes.

### Outcome assessment

The instrument development process was detailed in a previous paper (Nguyen et al., 2022). Briefly, instruments were selected by a larger collaborative group to align outcome instruments across multiple studies and to reflect multiple MHPSS domains. Consistent with the program’s focus on improving maternal psychosocial well-being and caregiver functioning and to align with the multi-study collaborative, primary outcomes were identified as maternal distress and functional impairment. Additional psychosocial outcomes also reflecting aspects of maternal well-being, including subjective well-being and coping, were included as secondary outcomes.

Standard translation and back-translation were used to develop a written version of the assessment battery in the Chittagonian Bengali dialect, which has a large degree of mutual intelligibility with the Rohingya dialect (which is not a written language); where the two languages diverged, the multilingual data collection team collaboratively decided on the appropriate Rohingya terms. Instruments were piloted and refined prior to baseline data collection, and final scale compositions were refined based on psychometric analyses (Nguyen et al., 2022). For all scales, questions employed a 2-week recall period and scores were generated as the mean of all contributing items to retain their original response range, with higher scores indicating greater magnitude of each domain (*e.g.*, greater distress, greater well-being).

#### Psychological distress

Psychological distress *(*primary) was assessed using an adapted instrument comprised of eight items from the Myanmar-wide version of the International Depression Symptom Scale (IDSS; Haroz et al., 2017) combined with the Kessler 6-item Psychological Distress Scale (K6; Kessler et al., 2010). Responses were based on the frequency of experience ranging from 0 “none” to 4 “all of the time.” The combined scale demonstrated excellent internal consistency at both baseline (*α* = .90) and follow-up (*α* = .92) and was treated as the primary outcome according to the approach described in the registered study protocol; however, given baseline findings that the IDSS items appeared to score higher than the K6 items (Nguyen et al., 2022), we also treated these as two separate instruments for which internal consistency was satisfactory to good (IDSS: *α* = .87 baseline, *α* = .91 follow-up; K6: *α* = .77 baseline *α* = .78 follow-up).

#### Functional impairment

Functional impairment (primary) was measured using the 12-item WHO Disability Assessment Schedule 2.0 (WHODAS; World Health Organization, 2010). Responses reflected difficulty in carrying out various activities, with options ranging from 0 “none” to 4 “extreme or cannot do.” Internal consistency was good (*α* = .81 baseline, *α* = .76 follow-up).

#### Subjective well-being

Subjective well-being (secondary) was assessed with a single-item Satisfaction With Life (SWL) rating (*i.e.*, “overall), as well as using six “domain-specific” items from the Personal Well-being Index (PWI; International Wellbeing Group, 2013). Responses for all items were provided on a range from 0 “no satisfaction at all” to 10 “completely satisfied.” The PWI demonstrated good internal consistency (*α* = .85 baseline, *α* = .88 follow-up).

#### Coping

Coping (secondary) was also assessed in two ways: with 10 items from the Brief COPE (B-COPE (Carver, 1997), and with four locally developed coping items (L-COPE; (Riley et al., 2017). These 14 items were retained from an initially larger pool and, diverging from the registered analysis plan, were treated as two separate scales based on psychometric analyses that indicated clearly distinct performance (Nguyen et al., 2022). Responses reflected the frequency of using each strategy, ranging from 0 “I haven’t been doing this at all” to 3 “I’ve been doing this a lot.” Internal consistency at baseline was good for both scales, although the L-COPE deteriorated at follow-up (B-COPE: *α* = .81 baseline, *α* = .87 follow-up; L-COPE: *α* = .76 baseline *α* = .61 follow-up).

### Blinding

By necessity, BFS providers and the field coordinator were aware of the intervention conditions based on which INCs received retraining. All US-based members of the research team remained blinded for the duration of the study, including during the initial analysis. Participants were not told what arm of the trial they were in, nor were the outcome assessors.

### Analyses

Analyses were conducted using Stata 17.0 (StataCorp, 2021). Baseline characteristics by group were summarized using cross-tabulations and summary statistics, reporting between-group differences as both raw and cluster-adjusted chi-square and t-tests (Herrin, 2002). Change scores were calculated by subtracting baseline from follow-up; improvement is indicated by a negative change score for the IDSS, K6 and WHODAS and a positive change score for the SWL, PWI and COPE scales. To examine within-group change, we entered each change score into a group-stratified linear regression model with cluster-robust standard errors. Consistent with our pre-registered analytic approach, between-group differences were analyzed using multilevel linear regression models to regress each change score onto a binary group variable while controlling for clustering by BFS site. As a sensitivity analysis, the models were rerun following McNeish and Stapleton’s (2016) recommendations for analyzing data within a small number of clusters, which included using a Kenward–Roger adjustment to fixed-effect standard errors (Kenward and Roger, 1997). All analyses were intention-to-treat. Analyses were led by AJN and SMM, who were blinded to study allocation.

### Sample size

Sample size calculations were conducted in optimal design to account for clustering within a two-level study with treatment at level 2 and outcomes assessed at level 1. Setting power at 80%, probability of a type 1 error at p = .05, and a small minimum detectable effect size of *d* = .3 on the primary study outcomes, we estimated a necessary sample size of approximately 250 individuals in each arm. The choice of a small effect size reflects the active comparison condition, which is likely to result in smaller between-group differences. Sample size was increased by 20% to account for potential loss to follow-up, resulting in a target sample size of n = 300 per arm.

## Results

### Sample description

**Figure 1.**
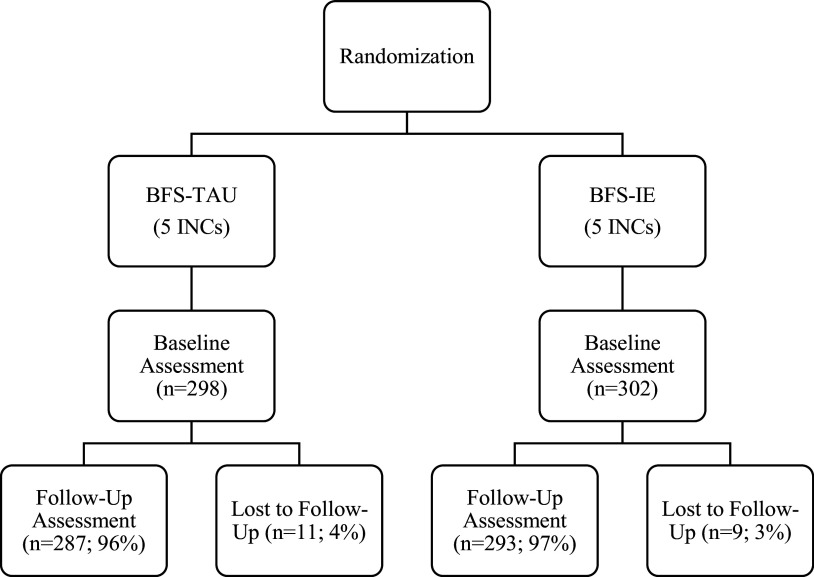
Consort diagram.

Recruitment and baseline data collection took place between November 2021 and January 2022. Follow-up data collection continued until March 2022. In total, 600 participants were recruited with approximately equal allocation across the study arms (Figure 1). The baseline demographic characteristics are shown in Table 3. Participants were all Rohingya-speaking women, largely in their 20s, who had been living in the camp on average for between 5 and 6 years (consistent with the 2017 refugee influx) and averaged approximately three children. A large majority of the women were currently breastfeeding, and approximately 11% of the sample were pregnant again at the time of intake. Their index children were predominantly girls. While women in the BFS-IE condition had been in the camps slightly longer, had slightly more children in the home on average and appeared to have had more access to at least some education and less regular access to meat, these differences were no longer statistically significant after adjusting for clustering. Among outcome variables, women in the BFS-IE condition entered the study with significantly higher mean B-COPE scores; no other baseline scores differed between conditions (Table 4).

**Table 3.** Characteristics of baseline sample

	BFS-TAU (*n =* 298)	BFS-IE (*n =* 302)	Total (*N =* 600)	Unadjusted *p*-value	Cluster-adjusted *p*-value*
	Mean (SD) or %	Mean (SD) or %	Mean (SD) or %		
Mother’s age in years	25.00 (4.79)	25.40 (5.04)	25.20 (4.92)	.315	.440
Years living in camp	5.31 (3.82)	6.14 (4.44)	5.73 (4.16)	.015	.554
Number of children	3.06 (1.59)	3.29 (1.81)	3.18 (1.71)	.103	.246
No. children in household	3.61 (1.72)	4.14 (1.88)	3.88 (1.82)	<.001	.201
No. adults in household	2.53 (1.59)	2.40 (.95)	2.47 (1.31)	.238	.528
Index child age in months	11.74 (4.47)	11.07 (4.44)	11.40 (4.46)	.067	.464
Index child sex					
Male	33.6%	35.4%	34.5%	.629	.721
Female	66.4%	64.6%	65.5%		
Mother’s education					
None	81.9%	66.9%	74.3%	<.001	.094
Some primary	11.4%	29.5%	20.5%		
Completed primary	6.7%	3.6%	5.2%		
Mother’s marital status					
Married	95.0%	96.7%	95.8%	.291	.477
Other	5.0%	3.3%	4.2%		
Mother’s employment status					
No employment	95.6%	97.0%	96.3%	.368	.499
Any employment	4.4%	3.0%	3.7%		
Household meat frequency					
Less than monthly	16.1%	31.1%	23.7%	<.001	.446
Monthly	60.4%	51.3%	55.8%		
Weekly	23.5%	17.6%	20.5%		
Currently pregnant					
No	89.3%	88.7%	89.0%	.839	.848
Yes	10.7%	11.3%	11.0%		
Currently breastfeeding					
No	10.9%	10.4%	10.7%	.835	.835
Yes	89.1%	89.6%	89.3%		

* From cluster-adjusted chi-square and *t*-tests for categorical and continuous variables, respectively.

**Table 4.** Within-group change and between-group difference-of-differences under primary analyses and adjusted estimation procedures

	BFS-TAU			BFS-IE			Difference-of-differences (ref: BFS-TAU)		REML estimate with K-R adjustment	
	Pre *(n =* 298*)*	Post *(n =* 287*)*	Change *(n =* 287*)*	Pre *(n =* 302*)*	Post *(n =* 293*)*	Change *(n =* 293*)*				
	Mean (SD)	Mean (SD)	Mean (SD)	Mean (SD)	Mean (SD)	Mean (SD)	Β (SE)	*p*–value	Β (SE)	*p*–value
IDSS/K6 combined	.85 (.68)	.70 (.75)	−.16 (.77)	.98 (.56)	.50 (.49)	−.48 (.64)*	−.30 (.16)	.058	−.30 (.18)	.129
IDSS	.97 (.77)	.81 (.86)	−.17 (.92)	1.08 (.65)	.53 (.58)	−.56 (.75)*	−.37 (.18)	.039	−.37 (.20)	.102
K6	.69 (.68)	.54 (.70)	−.16 (.72)	.85 (.54)	.47 (.45)	−.39 (.63)*	−.22 (.14)	.118	−.21 (.15)	.201
WHODAS	.52 (.45)	.34 (.35)	−.19 (.47)	.63 (.46)	.33 (.32)	−.30 (.40)*	−.10 (.12)	.393	−.10 (.14)	.468
SWL	7.27 (2.35)	7.34 (2.20)	.10 (2.39)	6.51 (1.98)	7.24 (1.54)	.76 (2.19)	−.09 (.66)	.891	−.09 (.74)	.906
PWI	6.94 (1.75)	7.28 (1.81)	.36 (1.51)	6.39 (1.34)	7.28 (1.04)	.92 (1.37)*	.58 (.28)	.038	.58 (.31)	.101
B–COPE	0.84 (.45)**	1.33 (.75)	.49 (.73)	1.27 (.53) ^*a*^	1.46 (.63)	.19 (.66)	−.32 (.29)	.276	−.32 (.33)	.358
L–COPE	2.32 (.63)	2.53 (.57)	.22 (.58)	2.29 (.66)	2.33 (.48)	.05 (.80)	−.19 (.24)	.424	−.19 (.26)	.494

* Significant (*p* < .05) within-group change.

** Significant (*p* < .05) between-group difference at baseline.

The 8-week follow-up completion was high, resulting in a sample of n = 580 (96.7% of baseline) for analysis. With the exception of one participant who suffered the loss of her child and another referred to higher care due to suicide risk, the primary reason for loss to follow-up was the inability to locate participants, potentially due to them moving or leaving the camp. No participants who were contacted at follow-up declined to be interviewed.

### Intervention exposure and fidelity

During their 2-month period in the study, women attended anywhere from 0 to 8 sessions in BFS-TAU and 0 to 5 sessions in BFS-IE, although their mean level of intervention exposure was similar (means of 2.85 and 2.50 sessions, respectively; p = .589). However, the format of the intervention did vary slightly; whereas women attended a similar number of individual BFS sessions across study arms (means of 1.79 and 1.99, respectively; p = .710), women in BFS-TAU attended marginally more group sessions than those in BFS-IE (1.05 *vs.* 0.5, p = .06).

Fidelity observations are described further elsewhere (see Le Roch et al., n.d.). Briefly, providers in intervention and comparison conditions demonstrated similarly high competency for nonspecific therapeutic skills and faced similar contextual challenges in achieving fidelity on some aspects of the intervention, especially preparing for and following up on the sessions. Where observations of fidelity did differ, they tended to favor the intervention group and reflected topics discussed in supervision, such as including a quick self-check, providing information on confidentiality, focusing on the session topic and using a metaphor to explain session objectives. Notably, although fidelity observers were initially blinded to the intervention condition, they were both quickly able to identify which sites were implementation-enhanced due to the activities they observed.

### Within-group change in outcomes

Pre, post and change scores for primary and secondary maternal outcomes are reported by group in Table 4. These results show that in both groups, differences in scores were in the direction of improvement (reduced symptoms of distress and functional impairment, improved subjective well-being and use of coping strategies). However, whereas the magnitude of the changes in the BFS-TAU group was smaller and not significantly different from zero, several statistically significant pre-post changes were observed in the BFS-IE group. Specifically, significant within-group improvements were observed for the combined IDSS/K6 measure of distress (mean = −.48, 95% CI: −.80, −.16; p = .014), as well as the two scales separately (IDSS mean = −.55, 95% CI: −.94, −.17; p = .016; K6 mean = −.39, 95% CI: −.62, −.15; p = .010); the WHODAS measure of functional impairment (mean = −.30, 95% CI: −.42, −,18; p = .002) and the six-item domain-specific PWI measure of subjective well-being (mean = .92, 95% CI: .35, 1.49; p = .011). An observed improvement in the SWL global well-being rating of slightly lower magnitude was not statistically significant (mean = .76, 95% CI: −.35, 1.86; p = .129); likewise, the magnitude of changes in coping was modest in size and not significant.

### Between-group change

Consistent with the within-group changes reported above, between-group difference-of-differences, where observed, favored the BFS-IE condition (Table 4). These included a significantly larger improvement in distress symptoms, a primary outcome, although this finding differed by measure. Specifically, whereas we pre-specified the combined IDSS/K6 scale as the primary outcome measure for distress, the magnitude of between-group difference in that scale was not statistically significant (mean = −.30, 95% CI: −.62, .01; p = .058). This appeared to reflect a divergence between the IDSS items, which as a standalone scale had higher baseline scores and statistically significant between-group change (mean = −.37, 95% CI: −.72, −.02; p = .039) and the K6, which showed both lower baseline scores and a smaller, nonsignificant degree of difference (mean = −.22, 95% CI: −.49, .05; p = .118). For the second primary outcome, functional impairment, no significant difference was observed by group.

Secondary outcomes likewise showed some divergence by measure. Whereas we observed a relatively strong and significant effect in subjective well-being as measured by the 6-item PWI (mean = .58, 95% CI: .03, 1.13; p = .038), no significant difference was observed for the global SWL rating (mean = −.09, 95% CI: −1.39, 1.20; p = .891). We also observed no significant differences in either measure of coping.

### Sensitivity analysis

Sensitivity models for the quantitative analysis with the Kenward–Rodger adjustment are also reported in Table 3. These results show that with relatively modest adjustments to the standard errors, none of the between-group differences remained statistically significant.

## Discussion

This study describes a hybrid implementation-effectiveness study of BFS, a PSS program for Rohingya refugee mothers of malnourished young children, in real-world conditions in Cox’s Bazar camps, Bangladesh. Mothers in both the BFS-TAU and BFS-IE demonstrated within-group improvement across a range of MHPSS outcomes. Comparisons of mothers between these two arms suggest added value for mothers attending a program where attention to implementation quality was intended to create a more efficient and effective program. The magnitude of within-group findings was consistent with a preventive intervention in which there were no baseline distress or impairment criteria for eligibility.

In this sample of participants, BFS was being offered in parallel to nutrition treatment that could also contribute to improved maternal well-being, both directly and through experienced improvements in the health of their children. Women in other settings have described the importance of their child’s growth and health to their mental health (Murray et al., 2017). Within the BFS-TAU condition, we cannot determine what proportion of the small observed improvements are due to BFS activities relative to other factors; however, as there were no other changes to nutrition services between the two groups, the greater improvements in the BFS-IE group support the added value of the BFS program. The smaller magnitude of between-group comparisons is also consistent with an active comparison group in which some level of improvement in the comparison condition attenuates the overall between-group effects. Noting the lack of a true control condition, it is important to consider both sets of comparisons together when evaluating the overall program impact. Qualitative findings reported elsewhere further highlight stakeholder perceptions of program impacts (Le Roch et al., 2023).

The difference in intervention between the two groups can largely be characterized in terms of differences in program training and implementation and supervision support. Whereas in the BFS-TAU condition, we continued to provide supportive services and supervision focused on program monitoring, those in the BFS-IE condition received additional training in more user-friendly materials that were designed to create a degree of standardization and ongoing clinical supervision over the subsequent months. Again, given that these additional implementation supports were offered as a package, it is difficult to determine the extent to which any observed changes are attributable to initial training relative to later supervision; however, as reported elsewhere, we saw changes in provider confidence after the training and prior to beginning supervision (Le Roch et al., n.d.). That said, both the observed drift in fidelity in our “as usual” comparison group, as well as broader lessons from the MHPSS field, reinforce the critical need for ongoing supervision to sustain high quality implementation (Kohrt and Bhardwaj, 2019; Lasater et al., 2020).

A strength of our study was our ability to leverage multiple measures for key outcomes of interest, including distress, subjective well-being and coping. Findings in each of these sets of outcomes present a complex picture that highlights the need to carefully attend to measurement in research. For example, whereas the K6 items reflect a more general construct of “distress,” the items we retained from the IDSS were more specific to symptoms of depression and had been more extensively adapted and tested for use with people from Myanmar. These differences may explain why the IDSS items appeared to be more sensitive, both in terms of higher baseline scores and capturing greater change over time. Likewise, whereas the PWI evaluated subjective well-being in terms of specific domains, differences between the PWI and the broader assessment of life satisfaction “overall” suggest that respondents may consider other important factors in their lives that were not represented on the PWI. Future research that is able to more thoughtfully operationalize subjective well-being for the particular culture and context would be a valuable contribution. However, we did attempt to bring in locally relevant coping items that were developed following qualitative work (Riley et al., 2017), and found that while participants endorsed these local coping strategies more highly, no significant changes in coping were observed when looking at either standard items or culturally specific items. As improved coping is a putative mechanism within the intervention’s theory of change, our inability to capture change here signifies a potential need for greater attention to contextualizing coping-focused PSSs within the program.

There are important limitations to be considered when the interpreting results from this study. First, given our study and funding timeline, we were restricted to a relatively brief follow-up period of eight weeks. Even in this relatively short time frame, we were unable to locate a small proportion of participants on account of the instability often characteristic of refugee settings. Due to this brief timeline, we cannot speak to the longer-term impacts of the program, either whether initially observed differences between the arms may diminish or if greater change may result with time.

Second, we randomized within pairs matched on only a few key features on account of the limited number of INCs available, rather than a simple randomization of all sites to one arm or the other. While we tried to carefully attend to rigor in our randomization process to minimize bias, unadjusted comparisons did highlight a few key characteristics that were not balanced across the two conditions, although clustering accounted for most of these differences.

Third, restrictions in program and research activities were enacted in response to the ongoing COVID-19 pandemic. In particular, the frequency of food distribution and nutrition appointments was limited to reduce potential COVID-19 exposure, and this led to families coming to the centers less frequently than they would have pre-pandemic. Thus, whereas often women are encouraged to attend weekly BFS sessions, in this study exposure to the intervention was more limited and individual meetings were promoted over group activities. Moreover, even in the arm with implementation supports, providers were observed to have low fidelity in some aspects of the intervention model, likely reflecting contextual challenges. While it is promising that we were able to see improvements over the intervention period even with a smaller dose, given the variation in delivery format, it is difficult to generalize these findings to other contexts or even this same context outside this particular period of time.

Finally, while there is no clear cutoff for a minimum number of clusters for multilevel modeling and the recommended minimum varies according to the parameters of interest, the potential for making inappropriate inferences increases when fewer clusters are compared (McNeish and Stapleton, 2016). When applying a more conservative adjustment to our statistical approach to account for the small number of clusters, point estimates of between-group comparisons in change in outcomes remained promising in magnitude but lost statistical significance. Our results for the effectiveness of the implementation enhanced *versus* as usual comparator BFS arm should therefore be interpreted with caution. The observed changes still have relevant implications for both the value of accessible, low-intensity PSSs overall and the critical importance of attending to implementation in the delivery of these programs.

Although the pragmatic, field-based approach to this research came with these limitations, the strength of this approach is the relevance of findings for improving practices in very challenging, real-world settings. Beyond the quantitative findings reported here, we have elsewhere reported improved confidence of providers in the BFS-IE condition, and notable differences in implementation between the two groups that were readily recognized by external fidelity observers (Le Roch et al., n.d.). During a time in which many services and social supports were likely disrupted in some fashion due to the COVID-19 pandemic, even the relatively modest BFS supports may have filled a critical gap in programming that was exacerbated during this period. Furthermore, this research demonstrates that small but feasible adjustments to implementation can both improve program delivery for maximizing impact and support effectiveness research.

Emergency health services or community outreach systems deliver essential care services to women and their children. Fundamental to life-threatening conditions and illnesses, however, those services rarely consider the well-being of the mothers and their babies, and each sector would address health issues around mother and child survival through its own scope of action and own set of interventions, rather than using an integrative approach that takes the mother–child relationship into consideration. Offering preventive and promotive interventions within the framework of BFS supports both maternal and child well-being. This work therefore aligns with current MHPSS research priorities not only with regard to systems integration, but through a focus on implementation, also meets priorities for better understanding effective workforce development and structures that can support implementation quality of what are often more flexible and broadly targeted programs (Tol et al., 2023). In particular, whereas more robust evidence exists for manualized psychotherapy programs, we demonstrate promising strategies to improve the quality of flexible, low-intensity PSSs with meaningful impacts on client outcomes in contexts affected by ongoing migration and/or instability.

## Conclusion

Relative to “as usual” intervention delivery that reflects typical programmatic drifts in fidelity, careful attention to a limited package of implementation enhancements appears to offer small but meaningful improvements in the overall intervention impact for participants. These findings must be considered within the study limitations that are typical of field-based research in challenging settings. However, taken together with participant- and provider-reported qualitative perceptions of enhanced impact (Le Roch et al., 2023) as well as provider and supervisor perceptions of increased competency (Le Roch et al., n.d.), these results highlight the critical need to attend to intervention quality and appropriate local contextualization even in the midst of challenging and rapidly changing humanitarian environments. In particular, it is notable that these improvements are not a result of increased dose of either intervention delivery at the participant level as reported here, or general supervision at the provider level (Le Roch et al., 2023), but rather the careful attention to quality and consistency in each of these components.

## Data Availability

The data that support the findings of this study are available *via* the MHPSS ISC Data Repository.
