# Supplementary material for: Psychosocial impacts of Baby Friendly Spaces for Rohingya refugee mothers in Bangladesh: A pragmatic cluster-randomized controlled trial
Source: Glob Ment Health (Camb). 2024 May 7;11:e64. doi: 10.1017/gmh.2024.58 (PMC11140488; doi:10.1017/gmh.2024.58)
Supplement: Peer review history

# Editor report: Recommendation: Psychosocial impacts of Baby Friendly Spaces for Rohingya refugee mothers in Bangladesh: A pragmatic cluster-randomized controlled trial — R0/PR2

**DOI:** 10.1017/gmh.2024.58.pr2

**Affiliations:** Community Health and Social Science, CUNY Graduate School of Public Health and Health Policy, United States

We have received two reviews, who has praised your study for its important contribution to the field and its transparent reporting of the methods and limitations. Reviewers have requested for additional background information (e.g., literature to support the rationale for the study), clarification and details in the methods section, particularly to emphasize the differences between the usual care and enhanced condition for BFS, as well as a few additional reflections and implications for practice and policy in the discussion section. Please respond to these points as well as our comments in your revised manuscript. Their suggestions and comments are listed below.

# Editor report: Decision: Psychosocial impacts of Baby Friendly Spaces for Rohingya refugee mothers in Bangladesh: A pragmatic cluster-randomized controlled trial — R0/PR3

**DOI:** 10.1017/gmh.2024.58.pr3

**Affiliations:** Johns Hopkins University Bloomberg School of Public Health, United States

No accompanying comment.

# Editor report: Recommendation: Psychosocial impacts of Baby Friendly Spaces for Rohingya refugee mothers in Bangladesh: A pragmatic cluster-randomized controlled trial — R1/PR5

**DOI:** 10.1017/gmh.2024.58.pr5

**Affiliations:** School of Government, Universidad de los Andes, Colombia

No accompanying comment.

# Editor report: Decision: Psychosocial impacts of Baby Friendly Spaces for Rohingya refugee mothers in Bangladesh: A pragmatic cluster-randomized controlled trial — R1/PR6

**DOI:** 10.1017/gmh.2024.58.pr6

**Affiliations:** Johns Hopkins University Bloomberg School of Public Health, United States

No accompanying comment.
